# Buruli ulcer community health education and medical screening in Ga South District, Ghana

**DOI:** 10.3389/fpubh.2025.1620853

**Published:** 2025-08-26

**Authors:** Charles A. Narh, Edwin Tetteh, NSPA-NMIMR, Lydia Mosi, Dorothy Yeboah-Manu

**Affiliations:** ^1^Noguchi Memorial Institute for Medical Research, University of Ghana, Legon, Ghana; ^2^West African Centre for Cell Biology of Infectious Pathogens, University of Ghana, Legon, Ghana; ^3^School of Medicine, Deakin University, Geelong, VIC, Australia; ^4^Department of Biochemistry, Cell and Molecular Biology, University of Ghana, Legon, Ghana

**Keywords:** community, health education and awareness, Ghana, Buruli ulcer, *Mycobacterium ulcerans*, screening, control programs and Noguchi

## Abstract

**Background:**

Buruli ulcer (BU) is caused by *Mycobacterium ulcerans* (MU), but the mode of transmission is unclear. BU starts as a nodule, which can progress to ulcer if not treated. Most of the affected seek help late due to socio-cultural beliefs of the cause of the disease, leading to long treatment course and disability. Therefore, to raise awareness about the disease and detect early forms of BU cases for treatment, the National Service Personnel Association of the Noguchi Memorial Institute for Medical Research (NSPA-NMIMR) conducted BU Community Health Education and Medical Screening (BU-CHEMS) in four endemic communities in the Ga South District of Ghana.

**Method:**

Between April–June 2010, the NSPA leadership conducted a series of seminars and media campaigns to raise public awareness about BU. Prior to the BU screening in the study communities, participants were shown BU documentaries to educate them and dispel myths about the disease. This was then followed by physical examinations for signs of BU; fine needle aspirates and/ or swabs were taken from nodules, plaques or ulcerative lesions, respectively, for laboratory confirmation of MU infection. Participants also volunteered for free medical screening - Body Mass Index (BMI, *N* = 58), blood pressure (*N* = 71) and blood group test (*N* = 424).

**Results:**

The media campaigns reached over 10 million people through national radio and TV, and the BU screening benefitted 2,500 participants. Most of the participants, 85%, were aware about the disease but not the cause. Of the 33 suspected cases identified with lesions (84.8% children), 78.8% were confirmed as positive for MU infection; representing 1,040 cases per 100,000 or 1% prevalence in the study population. All the confirmed cases commenced free BU treatment and were supported with medical supplies donated by NSPA-NMIMR to the Obom Health Centre, Ga South District. Participants with BMI ≥ 25 kg/m^2^ (overweight/obese, 43%) and hypertension (≥130 mmHg, 49%) received medical counselling.

**Conclusion:**

The BU-CHEMS program incentivized community participation to contribute to national BU control interventions and therefore can be further refined to complement activities of the National Buruli Ulcer Control Program (NBUCP).

## Introduction

Buruli ulcer (BU), a disease caused by *Mycobacterium ulcerans* (MU) is one of the neglected but treatable tropical diseases. MU belongs to the same genus as the bacteria that causes tuberculosis, but BU has received less attention (1). BU is endemic in more than 30 countries, occurring mostly in West Africa and Southeastern Australia (2). Although, the mode of transmission is unknown, studies in West Africa have associated infection with contact with MU-contaminated water bodies and in Australia, mosquitoes and possum were implicated (3–5). Infection leads to extensive destruction of skin and soft tissue with the formation of large ulcers usually on the legs or arms. In some endemic communities in Ghana, BU is associated with witchcraft and is known by local names such as “Odontihela” (describing the cotton wool appearance associated with the fatty necrosis), “Aboa gbonyo” (dreadful disease), and “Ashanti Asane” (meaning the boil disease originating from the Ashanti region) (6).

**Figure 1:**
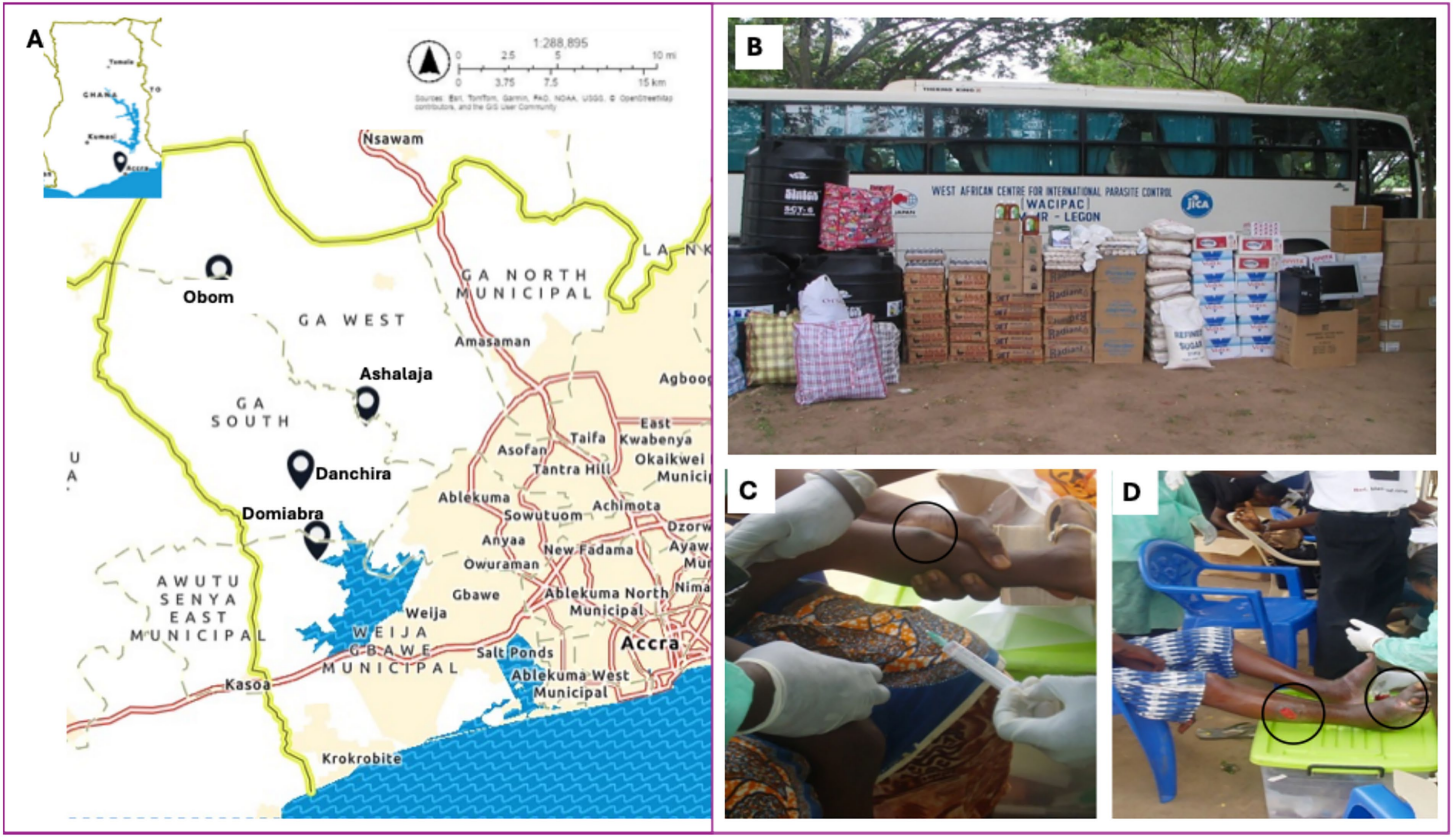
Buruli ulcer community health education and medical screening (BU-CHEMS). **(A)** Map of the study communities in the Ga South District of Ghana. **(B)** Medical items donated to Obom for the management of Buruli ulcer cases. Suspected BU patients with nodule **(C)** and multiple category III lesions **(D)**.

In Ghana, BU case load was highest, 115 cases in 100,000, i.e., 0.1%, in the Ashanti region in a 1999 study (6); and among suspected cases, the positivity rate declined from 76% in 2009 to 57% in 2010 (7). Public health control focus has largely been on early detection and treatment, which previously included complete excision of affected skin, followed by skin grafting to aid healing. Currently, BU is treated by long term antibiotic treatment and surgery, with skin grafts for ulcers to prevent permanent disability (8). Buruli ulcer treatment is free and health facilities such as the Ga West Municipal Hospital, Amasaman, Martins Catholic Hospital in Agroyesum, Amansie West District, and the Obom Health Centre (referred hereafter as Obom) in the Ga South District of the Greater Accra region (Figure 1A), offer routine free BU clinical services (9). Most endemic communities are rural and due to socio-cultural beliefs of the cause of BU, example, witchcraft (10), treatment seeking is considerably low among patients.

As part of the annual community engagement activities undertaken by the Noguchi Memorial Institute for Medical Research (NMIMR) and the activities of the Stop BU project (7), our group, the National Service Personnel Association (NSPA) comprising of 40 Research Assistants, conducted free BU Community Health Education and Medical Screening (BU-CHEMS) in four endemic communities in the Ga South District of Ghana.

## Methods

### Raising BU awareness through education and media campaigns

Between April–May 2010, the NSPA organized BU seminar series and media campaigns, with the aim of raising awareness about the disease and discuss areas that needed further research. These campaigns comprised a series of talks on all aspect of the disease, which were presented by the NSPA leadership, renowned BU researchers from NMIMR and the National Buruli ulcer Control Programme (NBUCP). The talks highlighted a critical need for community-led BU control interventions, research and development efforts to find cost-effective therapeutics including vaccines for the disease. During these campaigns, we solicited funds from corporate organizations and philanthropists to carry out the project (see supplementary data).

### Field work and BU-CHEMS

Between May–June 2010, we undertook community entry to seek permission from the community leaders (Chiefs and District Health Directorates) to conduct the project in four BU endemic communities – Domiabra, Ashalaja, Obom and Danchira in the Ga South District (Figure 1A). The project was carried out in two phases.

Phase A comprised BU educational campaigns to raise awareness about BU in the study communities, conducted from 21 to 22 July 2010; it involved showing WHO-certified BU documentaries to residents in the four communities. The documentaries (“video show”) were shown on large screens during the early evenings (6–9 pm) at the community market squares or playgrounds (>300 attendees per show per community). The documentaries were produced in English and therefore had to be interpreted in the local dialect – Ga language for easy understanding. The NSPA team engaged the services of eight community health workers and disease control officers to help mobilize the community for the BU documentaries and screening activities.

Phase B (Figures 1B–D) comprising general health checks and BU screening was conducted from 22 to 23 July 2010. Five marquees were mounted for BU screening and health checks (BMI, blood pressure/group tests). Participants were physically examined by the health physician and nurses for clinical signs of BU. Fine needle aspirates (FNA) of nodules/oedema (Figure 1C) and swabs of ulcers (Figure 1D) were taken from suspected BU cases for PCR confirmation at NMIMR using published protocols (11). The association donated medical supplies (Figure 1B and Supplementary material) to Obom to support clinical management of the BU patients. The NSPA team also administered paper-based questionnaires to collect participant data – demographics and knowledge/perceptions about BU. The following questions were asked; Have you ever heard about Buruli ulcer? Where did you hear it from? Do you know what causes Buruli ulcer? Will you go to the Health Center if you had Buruli ulcer? If a friend had Buruli ulcer, will you play or study with that person? The responses were entered and analyzed in Excel. Health check screening included body mass index (BMI), blood pressure and blood group testing.

### Project approval and ethics

The project was supported by NMIMR, NBUCP and Obom. These institutions provided written approval for the conduct of the project. The sample collection was embedded into a large BU study (Stop Buruli) and ethical clearance for the study was obtained from the institutional review board of the NMIMR (Federal-wide Assurance number FWA00001824) (7, 12). All participants provided verbal consent prior to participating in the health and BU screening, and written informed consent before their samples were taken for BU test confirmation. Children less than 18 years provided assent, and their parents consented. Refreshment was provided for all participants.

## Results

### Participant knowledge about BU

A total of 2,500 participants including 2,200 primary school children (<18 years) and 300 adults (≥ 18 years) were screened for BU based on physical examinations that looked for nodules, oedema, papules or ulcers. Due to logistical challenges, questionnaires were randomly administered to 50 participants; in summary, 85% of the respondents had heard about the disease (mainly from home or school). Over 50% of respondents had no idea of the cause of the disease. Among those who knew the cause, 85% attributed it to microorganisms while 15% attributed it to either witchcraft or punishment from a deity. Also, over 90% of the respondents said they would seek medical treatment. Regarding the question of social contact, 58% would not play with affected victims for fear of infection.

### Confirmed BU cases

Only the suspected cases (*N* = 33) had their FNA and or swab samples taken for laboratory confirmation (Table 1). Of these cases, 84.8% were children (<18 years) and 78.8% were confirmed as positive for MU infection; the majority, 73%, had single and CAT I legions including nodules (Figure 1C). Interestingly, all the Category III lesions were multifocal, predominantly on the lower limbs (Figure 1D). All the positive cases were followed up and scheduled to commence clinical treatment at Obom. In summary, the BU prevalence in the study population (*N* = 2,500) was 1,040 cases per 100,000 or 1%.

**Table 1:** Demographic characteristics of suspected BU patients.

Characteristics		No	Percentage
Age groups (Years)	0–17	28	84.8
	18–34	4	12.1
	Unknown	1	3.0
Gender	Male	19	57.6
	Female	14	42.4
Confirmed BU lesions	Positive	26	78.8
	Negative	7	21.2
Multifocality of lesions	Single	19	73.1
	Multiple	6	23.1
	Unknown	1	3.8
Size of lesions (cm)	CAT I (< 5)	19	73.1
	CAT II (5–15)	0	0.0
	CAT III (>15)	6	23.1
	Unknown	1	3.8

Health checks

**Figure 2:**
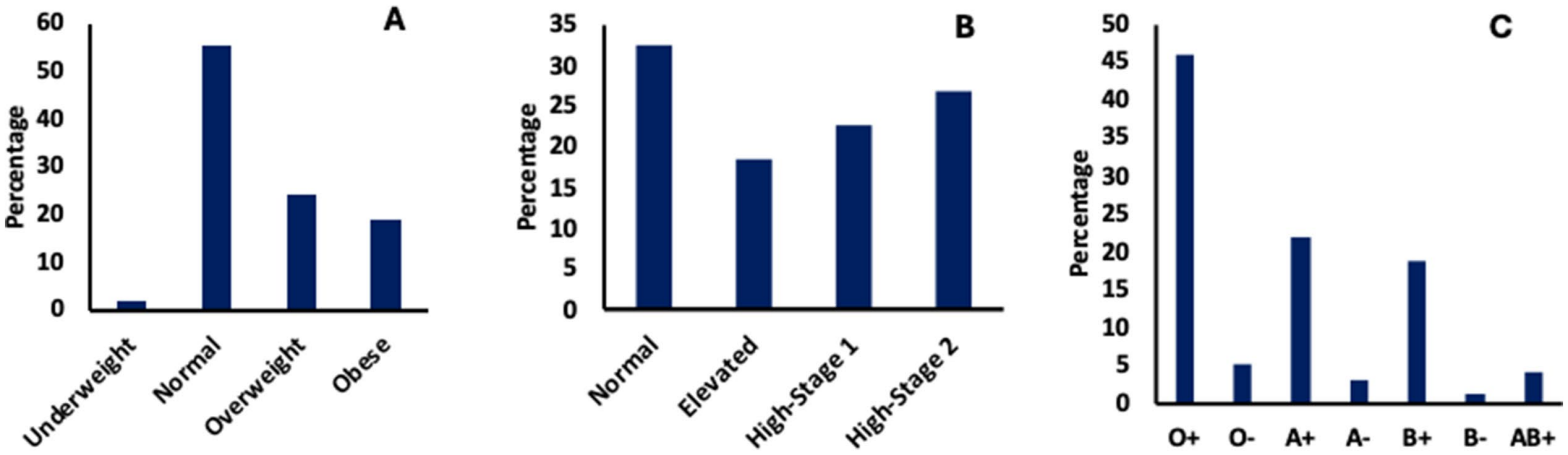
General medical screening. **(A)** Body mass index, **(B)** Blood pressure and **(C)** Blood group test.

BMI was categorized based on published standards (13). Of the 58 screened, the majority, 55%, had normal BMI (18.5–24.9 kg/m^2^) with 43% being overweight/obese, i.e., BMI ≥ 25 kg/m^2^ and 2% being underweight, <18.5 kg/m^2^ (Figure 2A). Of the 71 participants (age:18–90 years, median 35) that had blood pressure screening (Figure 2B), 32% were within normal ranges (90–120 mmHg), with 49% being considered hypertensive (≥130 mmHg) based on published standards (14, 15). There was no significant association between age, BMI and blood pressure (*p*-value ≥ 0.251). For the blood group test (Figure 2C), the majority, 46%, of the 424 participants screened had blood type O+. Participants had their readings explained to them and were given appropriate medical counselling and/or referred to Obom or their doctor.

## Discussion

This project aimed to educate and raise awareness about BU, and to conduct active search for cases in four endemic communities in the Ga South District. The WHO-certified BU documentaries we showed in the communities contained videos of BU patients with successful clinical treatments; this helped dispelled myths about the disease and encouraged the communities to participate in the screening program. Base on the 26 cases identified in our study population, the overall BU prevalence was estimated as 1,040 cases per 100,000 or 1%, which is higher than the 0.1% reported in 1999 (6), and the positivity rate among the suspected cases was 22% higher (78.8 versus 56.8%) compared to previous studies in 2010 (7). This underscores the need for continuous active case search, with laboratory confirmation. Most of the cases we identified had ulcers, which we referred to the Obom Health Centre for free treatment including wounding dressing. Thus, our program, which also donated medical items to support the management of BU cases contributed to NBUCP efforts to prevent disability among patients.

Control interventions have largely focused on clinical management including surgery to prevent permanent disabilities. Nearly all the cases (except one) we identified were ulcerative and without proper clinical care. Therefore, the Obom staff provided on-site wound dressing services before scheduling the patients for BU clinic at the Centre. Early clinical treatment including antibiotic therapy has been effective in preventing MU infections from progressing to the ulcerative stage (13, 16). The majority of the BU patients that we identified were children, with single category I ulcers, i.e., <5 cm in diameter. By providing early diagnosis and prompt treatment, our project helped to avert disfigurement, and potentially, permanent disability that might have developed as a result of scarring from the ulcers.

Our BU-CHEMS program focused on raising awareness about BU through social media campaigns, national radio and TV interviews (>10 million views), and engagement with the community through stakeholder meetings with community leaders and the NBUCP. The BU documentaries were translated in the local dialect, “Ga,” which made is easy for the community to understand BU including the mode of transmission, preventative measures and available clinical treatments. BU education and social interventions such as providing free transportation to patients to access clinical treatment are crucial to control efforts (12). The myths and stigmatization associated with BU are major barriers to patients accessing clinical care in Ghana. Example, 58% of respondents said they would not play with patients for fear of being infected. This may lead to patients not receiving adequate support to continue clinical treatment at home or manage mental health issues associated with scarring and disfigurement/disability (17). Our approach of delivering BU education in the local dialect and integrating free medical checks as part of the BU case searches were vital to incentivize community participation. Thus, BU-CHEMS can help improve existing control programs (18).

Routine health checks remain a luxury among rural populations in Ghana, partly due to the cost of service and accessibility to clinical expertise. For the majority of the participants this was their first time knowing their blood type, a crucial medical record for blood transfusion. For the BMI and blood pressure tests, we targeted adults (≥18 years), with the data showing that 55% of the participants had normal BMI and nearly half could be considered hypertensive. Notably, the underweight case we identified was 85-year-old with high blood pressure. For all cases, we provided appropriate medical counselling, including healthy lifestyle choices where needed, and referred all participants with hypertensive readings to see their doctor.

Due to logistical challenges/limitations, we could not collect data from all the 2,500 participants, which would have provided a large sample size to explore associations between BU and participant clinical/demographic data, in order to identify risk factors of infection and disease severity.

## Conclusion and recommendations

More education is needed to raise awareness about BU, and improved access to clinical diagnosis and treatments are crucial to BU control and elimination in Ghana. CHEMS can be integrated with current BU programs to increase community participation in BU control in endemic communities.

## Data Availability

The original contributions presented in the study are included in the article/Supplementary material, further inquiries can be directed to the corresponding author/s.

## Author’s note

This project dubbed “Buruli ulcer community health education and medical screening (BU-CHEMS) in Ga South District, Ghana” was carried out by the National Service Personnel Association of Noguchi Memorial Institute for Medical (NSPA-NMIMR), 2009/2010 batch with permission and support from the National Buruli Ulcer Control Programme (Korle-Bu Hospital), Noguchi memorial Institute for Medical, University of Ghana, Legon and the Obom Health Center.

## Group member of NSPA-NMIMR

Full membership list of the National Service Personnel Association of NMIMR (NSPA-NMIMR) is provided in the Supplementary material.
