# Supplementary material for: Buruli ulcer community health education and medical screening in Ga South District, Ghana
Source: Front Public Health. 2025 Aug 26;13:1620853. doi: 10.3389/fpubh.2025.1620853 (PMC12418778; doi:10.3389/fpubh.2025.1620853)
Supplement: Supplementary file 1 [file Supplementary_file_1.docx]

**Supplementary data**

**Sponsorship and transparency statement**

The association was able to donate items worth GH¢ 6,500 to the Obom Health Center. Much of the funds used for the project came from the Société Generale Ghana (SG-SSB) bank. The bank sponsored the association with GH¢ 5,000 and 50 branded SG-SSB T-shirts. Fueltrade Oil Ltd and Zoomlion GH Ltd donated GH¢1,000 each. Voltic GH Ltd gave the association 20 cartons of 500ml water and 20 bags of sachet water, PZ Cussons supported with some of their products and Tasty Treat Catering services gave the association take-away lunch packs for the 40 service personnel for two days. Additionally, NMIMR staff donated used clothes. Lastly, NSPA members contributed GH¢1,925 aside monthly dues for the project. The association also acknowledges contributions in cash and/or in-kind from staff of NMIMR towards the project.

**Medical items donated to the Obom Health Centre**

The medical supplies included gauze, Vaseline® gauze, gloves, Dettol®, soaps, cotton wool and methylated spirit. Other donations included bags of rice and sugar, oats, Milo®, biscuits and drinks, Voltic® bottled and sachet water, used clothes and a desktop computer. The NSPA also donated a Sintex polytank (to store drinking water) to each of the four schools and to the Obom Health Center. Confirmed positive cases were treated at the Health Centre.

**NSPA-NMIMR profile**

The National Service Personnel Association of Noguchi Memorial Institute for Medical Research (NSPA-NMIMR) is a recognized association in Noguchi for all national service personnel. It was formed by the 2007/2008 batch of service personnel. The association is governed by an executive board elected every year by the members. These includes the president, vice president, secretary, treasurer, organizer and the Head of Sponsorship who is elected by the other five executives after induction into office. The association also has a patron who sees to the general well-being of all the members and monitors matters of the association. Members pay monthly dues for the administrative running of the association. As part of our end of service celebration, the association carries out a charity project to help the less privileged in the society. Past projects have been medical screening for the inmates of the Ho Prisons (2008) and a Charity project at the Ho Leprosarium (2009). Our group 2009/2010 voted to undertake the BU-CHEMS following feasibility and endorsement by NMIMR administration.

Table 1: NSPA-NMIMR MEMBERSHIP 2009/2010

| **NAME** | **EXECUTIVE** | **DEPARTMENT** |
| --- | --- | --- |
| Constance A.Tsomafo |  | Animal Experimentation |
| Mary Eddy-Doh |  | Parasitology |
| Jennifer D.kwawukume |  | Electron Microscopy |
| Felix S.Amuzu |  | Chemical Pathology |
| Valentina Dornuki Ayim | Vice President | Electron Microscopy |
| Archer Priscilla |  | Epidemiology |
| Adubie Francis |  | IRB |
| Agyekum Jennifer O. |  | Parasitology |
| Amoah Owusu Yaw |  | Virology |
| Tetteh Edwin Richard | Head of Sponsorship | Accounts |
| Amoa-Bosompem Mildred |  | Virology |
| Tuffour Isaac |  | Nutrition |
| Mensah David Delali |  | Bacteriology |
| Voegborlo Selasi Vera |  | Parasitology |
| Narh Charles Akugbey | President | Parasitology |
| Motey Eva Emefa |  | Immunology |
| Samuel Ofori Addo |  | Bacteriology |
| Awalime Kwesi Dziedzom |  | Epidemiology |
| Baffour-Awuah Nana Yaa Anima | Organizer | Immunology |
| Aboagye James Odame |  | Virology |
| Phyllis Ofoe |  | Epidemiology |
| Esenam C.Afewu |  | Parasitology |
| Ibrahim Inna | Secretary | Bacteriology |
| Minta-Asare Keren |  | Virology |
| Eunice Dotse |  | Chemical Pathology |
| Dillis Adu-Harrison | Treasurer | Accounts |
| Agudey Dieudonne Kwame |  | Parasitology |
| Ahedor Believe |  | Animal Experimentation |
| Ashitey Pearl |  | Electron Microscopy |
| Atiemo Ofori Daniel |  | Accounts |
| Owusu-Ampaw Yeboah Yvonne |  | Chemical Pathology |
| Attafua Akua Akwaa |  | Parasitology |
| Osei-Kuffour Edmond |  | Parasitology |
| Ocran Rosalind Eugenia |  | Epidemiology |
| Tetteh Henry |  | Chemical Pathology |
| Arhin Irene Owusu |  | Immunology |

NSPA-NMIMR MEMBERSHIP 2009/2010 (CONT’D)

| Simpson Victoria Shirley |  | Bacteriology |
| --- | --- | --- |
| Tamim Madihah |  | Electron Microscopy |
| Emmanuel Blay Awusah |  | Library |
| Godfred Amo Agyekum |  | Parasitology |

Table 2: SPONSORSHIPS

| COMPANY/INSTITUTION | TYPE OF SPONSORSHIP |
| --- | --- |
| SG-SSB (main sponsor) | GH¢5,000 & 50 BRANDED SG-SSB T-  SHIRTS |
| FUEL TRADE CO. LTD | GH¢1,000 |
| ZOOMLION GH. LTD | GH¢1,000 |
| SOFAAMY ALUMINIUM CO. LTD | GH¢100 |
| TASTY TREATS CATERING SERVICES | LUNCH PACK FOR NSP |
| PZ CUSSONS | PRODUCTS |
| TV3 (Sunrise) | AIRTIME |
| CITI FM | AIRTIME |
| OBONU FM | AIRTIME |
| RADIO UNIVERS | AIRTIME |
| NMIMR | TRANSPORTATION/LOGISTICS/TRAINING |

CONTACT US

Noguchi Memorial Institute for Medical Research

College of Health Sciences

University of Ghana

P. O. Box LG 581

Legon-Accra

**Please note**: Charles Narh is now with Deakin University (charles.narh@deakin.edu.au
